# Identification of Two novel reassortant avian influenza a (H5N6) viruses in whooper swans in Korea, 2016

**DOI:** 10.1186/s12985-017-0731-7

**Published:** 2017-03-21

**Authors:** Jipseol Jeong, Chanjin Woo, Hon S. Ip, Injung An, Youngsik Kim, Kwanghee Lee, Seong-Deok Jo, Kidong Son, Saemi Lee, Jae-Ku Oem, Seung-Jun Wang, Yongkwan Kim, Jeonghwa Shin, Jonathan Sleeman, Weonhwa Jheong

**Affiliations:** 1 Environmental Health Research Department, National Institute of Environmental Research, Hwangyeong-ro 42, Seo-gu, Incheon, Republic of Korea; 2 U.S. Geological Survey, National Wildlife Health Center, Madison, WI USA

**Keywords:** H5N6, Highly pathogenic avian influenza viruses, Whooper swans, Korea

## Abstract

**Background:**

On November 20, 2016 two novel strains of H5N6 highly pathogenic avian influenza virus (HPAIVs) were isolated from three whooper swans (*Cygnus cygnus*) at Gangjin Bay in South Jeolla province, South Korea. Identification of HPAIVs in wild birds is significant as there is a potential risk of transmission of these viruses to poultry and humans.

**Results:**

Phylogenetic analysis revealed that Gangjin H5N6 viruses classified into Asian H5 clade 2.3.4.4 lineage and were distinguishable from H5N8 and H5N1 HPAIVs previously isolated in Korea. With the exception of the polymerase acidic (PA) gene, the viruses were most closely related to A/duck/Guangdong/01.01SZSGXJK005-Y/2016 (H5N6) (98.90 ~ 99.74%). The PA genes of the two novel Gangjin H5N6 viruses were most closely related to AIV isolates previously characterized from Korea, A/hooded crane/Korea/1176/2016 (H1N1) (99.16%) and A/environment/Korea/W133/2006 (H7N7) (98.65%). The lack of more recent viruses to A/environment/Korea/W133/2006 (H7N7) indicates the need for analysis of recent wild bird AIVs isolated in Korea because they might provide further clues as to the origin of these novel reassortant H5N6 viruses.

**Conclusions:**

Although research on the origins and epidemiology of these infections is ongoing, the most likely route of infection for the whooper swans was through direct or indirect contact with reassortant viruses shed by migratory wild birds in Korea. As H5N6 HPAIVs can potentially be transmitted to poultry and humans, continuous monitoring of AIVs among wild birds will help to mitigate this risk.

## Short report

As a natural reservoir for avian influenza viruses (AIVs), wild birds do not typically exhibit clinical signs of infection [1]. In most instances when AIVs of wild-bird origin are transmitted to poultry, infections are initially mild and represent a low pathogenic phenotype. Highly pathogenic AIVs (HPAIVs) arise following adaptation in domestic poultry, and some strains are known to cause significant illness or death if transmitted back to wild birds. Infection by HPAI H5N1 led to the deaths of thousands of wild birds at Lake Qinghai, western China in 2005 [2], and infection with HPAI H5N8 killed over a hundred wild birds of multiple species in the Republic of Korea in 2014/2015 [3].

On 16 November, 2016 the government of South Korea reported outbreaks of H5N6 HPAI in poultry farms in South Jeolla province [4]. On 20 November, 2016 one juvenile whooper swan (*Cygnus cygnus*) exhibiting neurological signs including torticollis and ataxia, along with the carcasses of two adults were found at Gangjin Bay in South Jeolla. Samples collected from all three birds were positive for H5 subtype AIV by RT-PCR [5]. To isolate the viruses, oropharyngeal and cloacal swabs, as well as tissue samples (trachea, liver, spleen and kidney) were inoculated into specific pathogen-free chicken eggs. Three isolates were identified as H5N6 AIVs by subtyping RT-PCR [6] and the entire genomes were determined [7]. No other AIV subtypes or Newcastle disease viruses were detected. The isolates from three whooper swans were designated as A/whooper swan/Korea/Gangjin 48/2016 (H5N6) (Gangjin 48, juvenile), A/whooper swan/Korea/Gangjin 49-1/2016 (H5N6) (Gangjin 49–1, adult) and A/whooper swan/Korea/Gangjin 49-2/2016(H5N6) (Gangjin 49–2, adult). The sequences of these isolates were deposited in GenBank with accession numbers < KY402046-KY402077 > .

The viruses contained conservative residues within the receptor-binding pocket of the hemagglutinin protein (HA, including Q226 and G228, H3 numbering), which is associated with a preference for “avian” type cell surface receptors containing alpha 2,3-sialic acid residues [8]. The viruses did not contain amino acid substitutions conferring resistance to adamantane and neuraminidase (NA) inhibitors [9]. The deduced amino acid sequence of the HA protease cleavage site contains a series of basic amino acid residues (LRERRRKR/GLF) characteristic of highly pathogenic AIVs. Experimental infection of 8-week old chickens by intravenous inoculation of the Gangjin 48 virus resulted in the deaths of all 8 birds within 25 h, conferring a intravenous pathogenicity index of 3.0. [5].

**Fig. 1:**
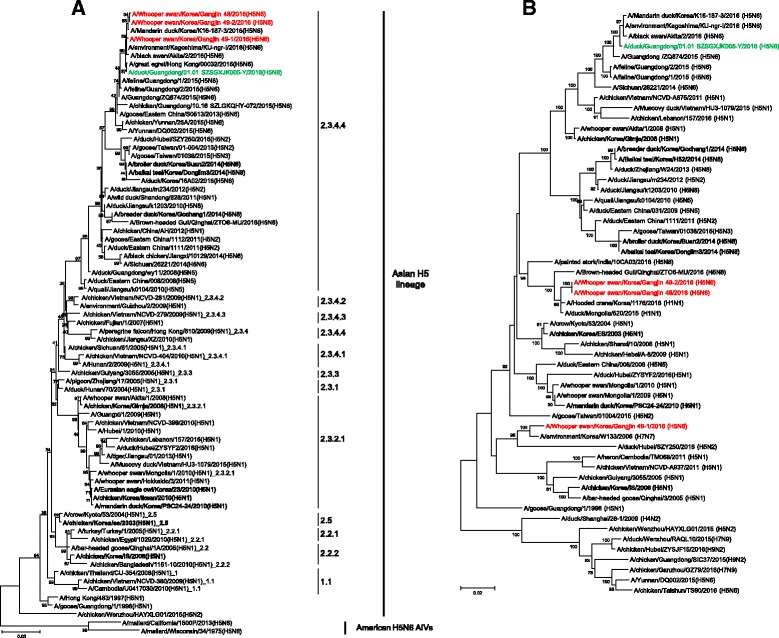
Phylogenetic tree of HA(**a**) and PA(**b**) genes of H5N6 viruses isolated from whooper swans. Clade 2.3.4.4 H5N6 HPAI isolates in Korea and Guangdong, Southern China in 2016 are shown in red and green, respectively. Phylogenetic trees were constructed using the maximum-likelihood algorithm of MEGA 6 with 1,000 bootstrap trials. Other HPAIVs detected in South Korea are indicated in boldface. The numbers at the nodes represent bootstrap values. The scale bar indicates the nucleotides substitutions per site

Phylogenetic analysis of HA gene sequences from the three viral isolates classified them within Asian H5 clade 2.3.4.4 lineage (Fig. 1a), which emerged in China during 2010–2011, and unlike earlier clades is known for a novel propensity to reassort with NA subtypes other than N1 [10]. The clade 2.3.4.4 H5N6 has been the prevalent lineage in Guangdong, Southern China since 2013 and spread to northeast China in 2014 [11]. Notably, the viruses are distinct from both groups A (A/Baikal teal/Korea/Donglim3/2014) and B (A/breeder duck/Korea/Gochang1/2014) of the 2.3.4.4 H5N8 viruses, and they are clearly distinguishable from H5N1 HPAIVs previously isolated in Korea, including (A/chicken/Korea/ES/2003 (clade 2.5), A/chicken/Korea/IS/2006 (clade 2.2), A/chicken/Korea/Gimje/2008 (clade 2.3.2.1), and A/mallard/Korea/1195/2010 (clade 2.3.2.1)) [3].

**Fig. 2:**
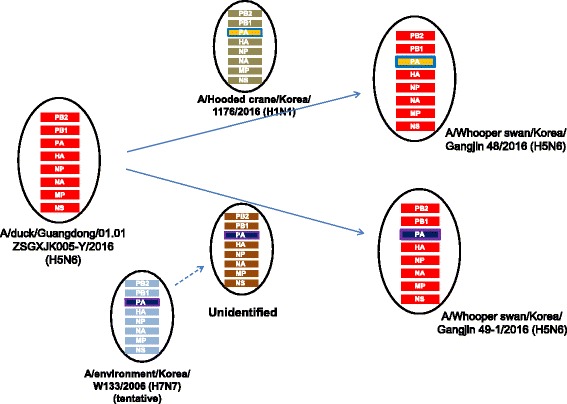
Schematic representation of the H5N6 virus strains. The eight gene segments are (indicated by horizontal bars starting from the top) PB2, PB1, PA, HA, NP, NA, M, and NS

Nucleotide identity analysis with BioEdit version 7.2.5 (http://bioedit.software.informer.com/) and Clustal Omega (http://www.ebi.ac.uk/Tools/msa/clustalo/) revealed that all three viruses were homologous with each other, sharing 99.03 ~ 100% nucleotide identity among seven out of eight segments (polymerase basic 2 (PB2), polymerase basic 1 (PB1), HA, nuclear protein (NP), NA, matrix (M), nonstructural (NS)). The PA subunit segments of Gangjin 48 and Gangjin 49–2 were identical to each other but differed (94.05% identity) from Gangjin 49–1, which suggests that at least two kinds of novel H5N6 HPAIV strains have circulated among wild birds in Korea. A BLAST (www.ncbi.nlm.nih.gov/genomes/FLU/FLU.html) search and phylogenetic analysis showed that all three viruses likely originated from A/duck/Guangdong/01.01SZSGXJK005-Y/2016 (H5N6, hereafter Guangdong 2016) (98.90 ~ 99.74% identity) (Fig. 2). The PA genes for Gangjin 48 and Gangjin 49–2 were closely related (99.16% identity) to A/hooded crane/Korea/1176/2016 (H1N1), while the PA gene of Gangjin 49–1 was more closely related (98.65% identity) to A/environment/Korea/W133/2006 (H7N7) (Fig. 1b). The data does not preclude the possibility that a as yet unidentified, but more contemporary, virus might be the actual donor of the PA gene to Gangjin 49–1 (Fig. 1b).

Whooper swans breed in northern Eurasia and winter in Europe and eastern Asia, including South Korea. Previous satellite data describing their southern migration to South Korea indicate the birds follow the Flyway along inner Mongolia, northeast China and the Korean peninsula during October and November [12]. Indeed, on 27 November many migratory bird species including whooper swans (>1,700), mallards (*Anas platyrhynchos*, > 3,500) and spot billed ducks (*Ana poecilorhyncha*, > 1,500) were observed around Gangjin Bay.

It is possible that whooper swans introduced both H5N6 viruses into Korea. However, it is also possible that whooper swans which are highly susceptible to HPAI H5N1 infection [13], may have been exposed locally through direct or indirect contact with other H5N6-infected but asymptomatic migratory species, such as mallards or spot-billed ducks, or perhaps through exposure to infected poultry [14]. Wild bird species differ in their response to HPAIV infection, and it is likely that unidentified asymptomatically-infected migratory birds could have introduced an ancestral virus, similar to Guangdong 2016, into Korea. Moreover, these or other species of wild birds could then play a role in generating novel reassortant viruses when co-infected with other AIVs. Indeed, our research group has isolated more than sixty AIVs from wild birds and waterfowl feces since October 2016 (unpublished data). Genetic analysis of these AIVs might provide clues as to the origin of these viruses; the characterization of which will be further detailed in a subsequent publication.

In conclusion, we have identified the two novel HPAIV H5N6 strains from three whooper swans in Korea. Phylogenetic and genetic analyses have shown that the two strains resulted from reassortment of the clade 2.3.4.4 H5N6 viruses that circulated in Guangdong, and they are distinguishable from HPAIVs that caused previous poultry and wild bird outbreaks in Korea. Increased understanding of clade 2.3.4.4 H5N6 virus transmission and pathogenesis in wild birds would serve to increase knowledge of the risk these viruses present to wildlife, domestic animals, and humans. Furthermore, as the H5N6 HPAIVs are potentially transmissible to poultry and humans, continuous monitoring of AIVs among wild birds is warranted.

## Abbreviations

AIVs: Avian Influenza Viruses
HA: Hemagglutinin protein
HPAIVs: Highly Pathogenic Avian Influenza Viruses
M: Matrix protein
NA: Neuraminidase protein
NP: Nuclear protein
NS: Nonstructural protein
PA: Polymerase Acidic protein
PB1: Polymerase Basic 1 protein
PB2: Polymerase Basic 2 protein
RT-PCR: Reverse Transcriptase-Polymerase Chain Reaction
